# Association of antihypertensives and Parkinson’s disease in a primary care population matched for underlying diagnosis

**DOI:** 10.1371/journal.pone.0299985

**Published:** 2024-03-20

**Authors:** Anette Schrag, Karel Kostev

**Affiliations:** 1 Department of Clinical and Movement Neurosciences, UCL Queen Square Institute of Neurology, University College London, London, United Kingdom; 2 University Clinic, Philipps-University, Marburg, Germany; 3 Epidemiology, IQVIA, Frankfurt, Germany; HT Ong Heart Clinic, MALAYSIA

## Abstract

**Purpose:**

To examine the association of several antihypertensive medication classes with incidence of Parkinson’s disease (PD), taking into account possible underlying conditions.

**Methods:**

In a case-control study based on a large primary care database and including 21,981 PD cases and 21,981 non-PD controls matched for age, sex, and possible treatment indications associations with different antihypertensive medication groups, including diuretics, betablockers, calcium channel blockers, angiotensin-converting enzyme inhibitors and angiotensin-II receptor-blockers and PD were examined.

**Results:**

Antihypertensive medications overall were associated with a lower risk of subsequent diagnosis of PD (OR: 0.94, 95% CI 0.90–0.97), with the negative association most significant for medications acting on the renin–angiotensin–aldosterone system. A positive association with diagnosis of PD was only seen for betablockers and restricted to those with relatively young age and not in those with longer treatment duration.

**Conclusion:**

When taking into account underlying diagnoses, antihypertensive medications overall were associated with a reduced incidence of PD.

## Introduction

The risk of developing Parkinson’s disease (PD) has been reported to be decreased with antihypertensive medications, particularly angiotensin-converting enzyme inhibitors (ACEi) and angiotensin-II receptor-blockers (ARB) [1, 2], as well as calcium channel blockers (CCB) [3–5]. Betablockers (BB) have been suggested to be associated with an increased risk of PD in the short term [6], thought to be due to inverse causality, and decreased risk in the long term [6, 7]. However, previous studies have largely examined individual medication groups, making it difficult to compare between the associations of PD with different antihypertensives. Furthermore, these studies have largely not adjusted for the underlying condition, and e.g. hypertension itself has also been associated with an increased risk of PD [8–10] and rate of other cardiovascular or renal diseases may also differ but has not been clearly determined. The differential associations of these conditions and their treatments with PD is important in order to determine the potential effect of medications.

We here undertook an analysis of different antihypertensive medications’ associations with later diagnosis of PD, taking into account the underlying diagnosis.

## Methods

### Database

This case control study used data from German primary care practices from the Disease Analyzer database (IQVIA). Details of the methodology have been published previously [11]. In brief, the Disease Analyzer database contains data on demographic variables, diagnoses and prescriptions obtained in general and specialized practices in Germany. The quality of the data is assessed every month based on several criteria (e.g., completeness of documentation and linkage between diagnoses and prescriptions). This database covers approximately 3–6% of all private practices in Germany. It has previously been shown that the panel of practices included in the Disease Analyzer database is representative of general and specialized practices in Germany [11–13]. The database has already been used in previous studies focusing on PD. One of us has previously examined associations of antihypertensive medications with diagnosis of PD previously, but without direct matching by underlying diagnosis [14].

### Study population

**Fig 1:**
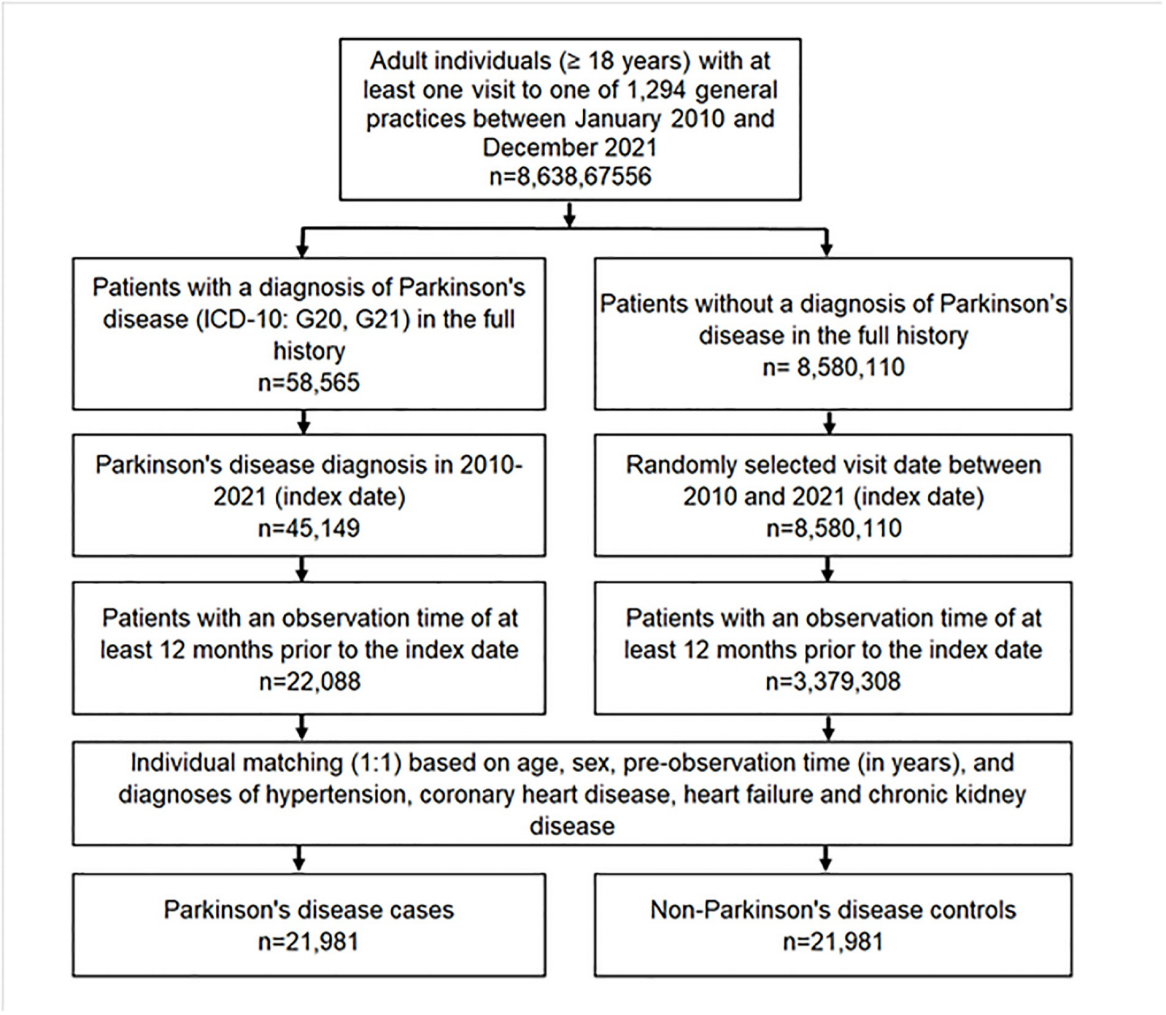
Selection of study patients.

The study population included all patients aged ≥18 years with a diagnosis of PD (ICD-10 code: G20 between January 2010 and December 2021 (index date) who had at least one year of observation prior to the index date. Controls were patients without PD who were matched (1:1) by age, sex, their pre-diagnostic observation time in years, and diagnoses documented prior to the index date including hypertension (ICD-10: I10), chronic ischemic heart disease (ICD-10: I25), heart failure (ICD-10: I50), and chronic kidney disease (ICD-10: N18, N19). For individuals without PD, the index date was a randomly selected visit date between January 2010 and December 2021. The flow diagram of study participants is shown in Fig 1.

### Study outcome

Outcome of the study was the association between prescriptions of antihypertensive drugs and subsequent PD diagnosis. Exposure was prescription of antihypertensive drugs, with classification based on the anatomical therapeutic chemical classification system (ATC) by the European Pharmaceutical Market Research Association (EphMRA), into diuretics (DIU) (ATC: C03, C07B1, C08B1, C09B1, C09D1), betablockers (BB) (ATC: C07, C08B2, C09B2, C09D2), calcium channel blockers (CCB) (ATC: C08, C09B3, C09D3), angiotensin-converting enzyme inhibitors (ACEi) (ATC: C09A, C09B, C09D4), and angiotensin II receptor blockers (ARB) (ATC: C09C, C09D, C07B2).

### Statistical analyses

Demographic and clinical characteristics of cases and controls after 1:1 propensity-score matching were evaluated using the Wilcoxon signed-rank test for continuous variables, the McNemar test for categorical variables with two categories, and the Stuart-Maxwell test for categorical variables with more than two categories. Associations between diagnosis of PD (dependent variable) and previous antihypertensive drug prescriptions (separately DIU, BB, CCB, ACEi, ARB) were examined using a multivariable logistic regression model. We conducted regression models for total study population and separately for four age groups, women and men. Bonferroni adjustment for multiple comparisons resulted in p-values <0.007 as significance cut-off. In order to understand associations depending on treatment duration, sensitivity analyses were conducted with restriction to those with a minimum duration of at least one, three and five years.

## Results

After 1:1 matching for demographics and diagnoses, 21,981 PD cases and 21,981 non-PD controls were available for analyses. Mean age at the index date was 76.4 (SD 10.3) years and 46.8% were female in both groups. On average, both cases and controls had 8.9 (SD 6.3) years of pre-observation time prior to index date. Furthermore, hypertension (70.5%) was the most frequent diagnosis, following by chronic ischemic heart disease (28.1%); heart failure (18.4%) and chronic kidney disease (14.5%; Table 1).

**Table 1:** Characteristics of study patients after 1:1 matching.

Variable	Parkinson’s disease (n = 21,981)	No Parkinson’s disease (n = 21,981)	P-value
*Age (in years)*			
Mean (standard deviation)	76.4 (10.3)	76.4 (10.3)	1.000
≤ 60	1763 (8.0)	1763 (8.0)	1.000
61–70	2965 (13.5)	2965 (13.5)	
71–80	8709 (39.6)	8709 (39.6)	
>80	8544 (38.9)	8544 (38.9)	
Sex			
Female	10279 (46.8)	10279 (46.8)	1.000
Male	11702 (53.2)	11702 (53.2)	
Observation time prior to the index date (year), mean (standard deviation)	8.9 (6.3)	8.9 (6.3)	1.000
*Conditions documented prior to or at the index date* *			
Hypertension	15485 (70.5)	15485 (70.5)	1.000
Heart failure	4039 (18.4)	4039 (18.4)	1.000
Chronic ischemic heart disease	6168 (28.1)	6168 (28.1)	1.000
Chronic kidney disease	3195 (14.5)	3195 (14.5)	1.000

Data are absolute numbers (%) unless otherwise specified.

*Patients can have more than one comorbid disorder at the same time

In a multivariate analysis of the overall population, diagnosis of PD was associated with a reduced risk of all antihypertensive medications except diuretics (Table 2). ARB were associated with a lower risk of PD diagnosis overall, particularly in the age groups 71–80 years, and in both women and men; the negative association for ACEi and for CCB was significant in the overall analysis only, and BB had a negative association with PD in the overall population and in the age group over 80 years, but a positive association with subsequent PD diagnosis in the population younger than 60 years.

**Table 2:** Association between antihypertensive drug prescriptions (ever use versus never use) and subsequent Parkinson’s disease total and by age group.

Population	Proportion (%) in patients with PD	Proportion (%) in patients without PD	Crude OR (95% CI)	P-value*	Adjusted OR (95% CI)	P-value*
*Total population*						
DIU	12.9	12.8	1.01 (0.95–1.06)	0.820	1.06 (1.01–1.12)	0.013
BB	10.2	11.4	0.89 (0.84–0.95)	<0.001	0.94 (0.89–0.98)	0.003
CCB	8.6	9.7	0.88 (0.82–0.93)	<0.001	0.93 (0.88–0.97)	0.002
ACEi	10.8	11.7	0.92 (0.87–0.97)	0.005	0.91 (0.87–0.95)	<0.001
ARB	5.9	7.1	0.82 (0.76–0.88)	<0.001	0.89 (0.85–0.94)	<0.001
Age ≤ 60						
DIU	6.7	6.6	1.02 (0.78–1.33)	0.892	0.97 (0.73–1.28)	0.823
BB	10.5	7.5	1.45 (1.15–1.83)	0.002	1.48 (1.16–1.89)	0.002
CCB	4.5	4.9	0.93 (0.68–1.27)	0.634	0.91 (0.65–1.26)	0.560
ACEi	8.5	7.2	1.20 (0.94–1.53)	0.151	1.18 (0.91–1.54)	0.210
ARB	3.0	4.1	0.72 (0.50–1.03)	0.071	0.70 (0.48–1.03)	0.067
Age 61–70						
DIU	11.1	9.9	1.14 (0.96–1.34)	0.128	1.24 (1.04–1.47)	0.018
BB	10.1	10.6	0.95 (0.80–1.12)	0.552	0.97 (0.81–1.15)	0.693
CCB	7.1	8.3	0.85 (0.70–1.03)	0.089	0.86 (0.71–1.05)	0.136
ACEi	9.9	11.2	0.85 (0.72–1.01)	0.062	0.82 (0.69–0.98)	0.032
ARB	5.6	6.6	0.83 (0.67–1.03)	0.094	0.82 (0.66–1.02)	0.079
Age 71–80						
DIU	12.5	12.6	0.99 (0.91–1.08)	0.819	1.06 (0.97–1.17)	0.197
BB	9.6	11.1	0.85 (0.77–0.94)	0.002	0.88 (0.80–0.97)	0.012
CCB	8.7	10.1	0.86 (0.76–0.95)	0.003	0.89 (0.80–0.99)	0.033
ACEi	10.7	11.8	0.90 (0.82–0.99)	0.026	0.92 (0.83–1.01)	0.078
ARB	6.1	7.5	0.80 (071–0.90)	<0.001	0.82 (0.72–0.92)	0.001
Age >80						
DIU	15.2	15.3	0.99 (0.91–1.08)	0.798	1.05 (0.96–1.15)	0.278
BB	10.9	12.7	0.84 (0.76–0.92)	<0.001	0.85 (0.77–0.94)	0.001
CCB	9.9	10.9	0.90 (0.81–0.99)	0.029	0.92 (0,83–1.02)	0.115
ACEi	11.7	12.6	0.92 (0.84–1.01)	0.083	0.94 (0.85–1.03)	0.183
ARB	6.4	7.6	0.84 (0.75–0.94)	0.003	0.86 (0.76–0.97)	0.012
Women						
DIU	13.3	12.9	1.03 0.95–1.12)	0.445	1.10 (1.01–1.20)	0.032
BB	10.5	11.5	0.90 (0.83–0.98)	0.020	0.92 (0.84–1.01)	0.065
CCB	9.5	10.8	0.87 (0.80–0.97)	0.003	0.90 (0.82–0.98)	0.021
ACEi	11.0	11.9	0.92 (0.84–1.00)	0.049	0.92 (0.84–1.01)	0.067
ARB	6.3	7.6	0.81 (0.73–0.90)	<0.001	0.82 (0.73–0.92)	<0.001
Men						
DIU	12.5	12.7	0.98 (0.91–1.06)	0.679	1.04 (0.96–1.13)	0.322
BB	10.0	11.3	0.88 (0.81–0.96)	0.002	0.90 (0.83–0.98)	0.016
CCB	7.8	8.8	0.88 (0.80–0.97)	0.007	0.91 (0.83–1.00)	0.050
ACEi	10.6	11.4	0.92 (0.85–1.00)	0.041	0.93 (0.86–1.02)	0.106
ARB	5.5	6.7	0.82 (0.74–0.91)	<0.001	0.83 (0.74–0.93)	0.001

Abbreviations: OR odds ratio; CI confidence interval.

*Bonferroni adjusted p-value 0.007

When only taking into account those with treatment durations over one, three and five years, for diuretics stronger negative associations with a subsequent diagnosis of PD were seen in the longer treatment duration groups, whereas for CCB treatment durations of at least one year and for ARB and ACEi only treatment durations of at least five years were negatively associated with negatively diagnosis of PD (Table 3). BB treatment was no longer negatively associated with diagnosis of PD in any of the treatment duration longer than one year.

**Table 3:** Association between antihypertensive drug prescriptions (depending on therapy duration) and subsequent Parkinson’s disease.

Population	Crude OR (95% CI)	P-value*	Adjusted OR (95% CI)	P-value*
At least 1 year of therapy				
DIU	0.93 (0.90–0.98)	0.003	0.94 (0.90–0.99)	0.009
BB	0.96 (0.92–1.00)	0.049	0.95 (0.91–0.99)	0.019
CCB	0.93 (0.89–0.98)	0.003	0.91 (0.87–0.96)	<0.001
ACEi	0.95 (0.91–0.99)	0.017	0.94 (0.90–0.98)	0.004
ARB	0.95 (0.91–1.00)	0.045	0.95 (0.90–1.00)	0.046
At least 3 years of therapy				
DIU	0.93 (0.89–0.98)	0.004	0.90 (0.86–0.95)	<0.001
BB	0.99 (0.95–1.04)	0.642	0.98 (0.93–1.03)	0,446
CCB	0.95 (0.90–1.01)	0.106	0.94 (0.88–1.00)	0.035
ACEi	0.95 (0.90–1.00)	0.035	0.93 (0.89–0.98)	0.011
ARB	1.02 (0.95–1.08)	0.605	1.02 (0.96–1.09)	0.568
At least 5 years of therapy				
DIU	0.91 (0.86–0.96)	<0.001	0.88 (0.83–0.93)	<0.001
BB	1.02 (0.96–1.07)	0.592	1.01 (0.95–1.07)	0.696
CCB	0.96 (0.89–1.03)	0.222	0.94 (0.88–1.02)	0.121
ACEi	0.92 (0.87–0.98)	0.008	0.91 (0.86–0.97)	0.003
ARB	1.12 (1.03–1.21)	0.006	1.13 (1.04–1.22)	0.005

Abbreviations: OR odds ratio; CI confidence interval.

*Bonferroni adjusted p-value 0.007

## Discussion

In this study antihypertensive medications overall were associated with a lower risk of subsequent diagnosis of PD, with the negative association most significant for medications acting on the renin–angiotensin–aldosterone system including ACEi and ARB, followed by CCB. A positive association with diagnosis of PD was only seen for betablockers and restricted to those with relatively young age and not seen in those with longer treatment duration, suggesting that this is a reverse effect based on initial treatment of tremor in younger patients with betablockers. On the other hand, in older patients there was also a negative association with diagnosis of PD with betablockers. Diuretics, which have not previously been reported to be associated with reduced risk of PD, were negatively associated with the diagnosis in all groups that had been on treatment for longer than one year. A suggestion of a slight increase in incidence with diuretics was only seen when adjusted for prescription of other antihypertensives and was not significant when controlled for multiple comparisons. This was reversed when excluding those with a duration of use of diuretics of less than one year. It is possible that diuretics are more commonly used shortly before a formal diagnosis of Parkinson’s is made, potentially through increased medical attention at this time.

These results are keeping with other reports on database analyses, including a study in patients with newly diagnosed hypertension who had a substantially reduced risk of PD over a 8.4 year time period, after adjusting for a number of variables [15]. Our results were supportive of this association, although the effect size was much smaller. In contrast to our study, on the other hand, a recent study using a mendelian randomization approach did not find an association of antihypertensive drug groups and incident PD [4]. However, this analysis was based on assumption of blood pressure being the mediator for the relationship with PD, and it is therefore possible that the mechanism of action for a risk reduction of PD with antihypertensive medications is different from the effect of blood pressure.

Our matching not only for age, sex and pre-diagnostic observation period but also for possible underlying indications allowed for a reduction in confounding effects on the associations due to these diagnoses, revealing a more consistent negative association with subsequent diagnosis of PD across antihypertensive medications including diuretics in those treated for more prolonged periods. This suggests that at least some of the observed negative associations are due to a generic effect not related to specific compound mechanisms but rather to an indirect effect eg on the vascular system [16]. Nevertheless, there were difference between antihypertensive groups which may point to additional specific effects on pathogenetic mechanisms in PD. For example, ARB have been reported to decrease dopaminergic neuronal loss and reduced oxidative stress in PD by influencing mitochondrial function [1, 2], whereas other mechanisms such as for CCB are poorly understood [17].

### Limitations

Despite significant negative associations in antihypertensives overall, not all sub-analyses reached significance and there was variation between groups. It is however possible that our study was not large enough to detect significant associations that did not reach our predefined threshold. We may also have missed diagnoses that were prescribed in secondary care only and it is possible that there are other risk factors for PD that may represent further confounding factors that influence the association between antihypertensives, A further limitation is lack of information on the severity of baseline diagnoses like hypertension or heart failure, and on smoking behavior, alcohol consumption, and other life-style factors. Finally, it is also possible that different types of antihypertensive subclasses have an additional effect that may not be captured in the analysis of the overall class, e.g. amongst dihydropyridine but not non-dihydropyridine and between centrally and peripherally acting CCB have been associated with a reduced risk of PD [17].

In summary, however, when accounting for underlying diagnoses, antihypertensives were overall negatively associated with subsequent diagnosis of PD. The positive association with betablockers seen in patients under 60 years was not seen in analyses of those with longer treatment duration. Future studies should study potential mechanisms and ensure that underlying potential confounding diagnoses are adjusted for.

## Supporting information

S1 Data Minimal underlying dataset of antihypertensive use. (XLSX)
